# Infarct size is overestimated by contrast-enhanced CMR in the acute phase but not at 7 days when compared with histopathology

**DOI:** 10.1186/1532-429X-16-S1-O67

**Published:** 2014-01-16

**Authors:** Robert Jablonowski, Henrik Engblom, Mikael Kanski, David Nordlund, Sasha Koul, Jesper van der Pals, Einar Heiberg, David Erlinge, Marcus Carlsson, Håkan Arheden

**Affiliations:** 1 Dept. of Clinical Physiology, Lund University, Lund University Hospital, Lund, Sweden; 2 Dept. of Cardiology, Lund University, Lund University Hospital, Lund, Sweden

## Background

Late gadolinium enhancement (LGE) cardiac magnetic resonance (CMR) is considered the gold standard for quantification of myocardial infarction in vivo. Myocardium with increased fractional distribution volume (fDV) due to acute necrosis or chronic scar exhibit hyperenhancement relative to viable myocardium when using a gadolinium-based extracellular contrast agent. Previous studies have shown conflicting results regarding the existence of a reversibly injured peri-infarction zone (PiZ) early after acute myocardial infarction (MI). Therefore, the aim of this experimental animal study was to measure infarct size in relation to histopathology acute and 7 days after MI and relate these findings to assessment of relative fDV in different parts of the myocardium.

## Methods

A total of 17 pigs were subjected to a 40-minute LAD occlusion followed by 6 hours (n = 9) or 7 days (n = 8) of reperfusion. Gadolinium-DOTA was injected for in vivo and high resolution ex vivo MI quantification by CMR. In addition, a radioactive tracer (^99 m^Tc-DTPA) was injected for determination of relative fDV (relative to normal myocardium) using quantification of radioactive counts in tissue samples taken from the myocardium at risk (MaR), the PiZ and the infarct core. Infarct size was also assessed by triphenyltetrazolium chloride (TTC) staining. Finally, the TTC stained slices of the ex vivo heart with TTC signs of infarction were re-scanned to enable a true slice-by-slice comparison.

## Results

**Figure 1:**
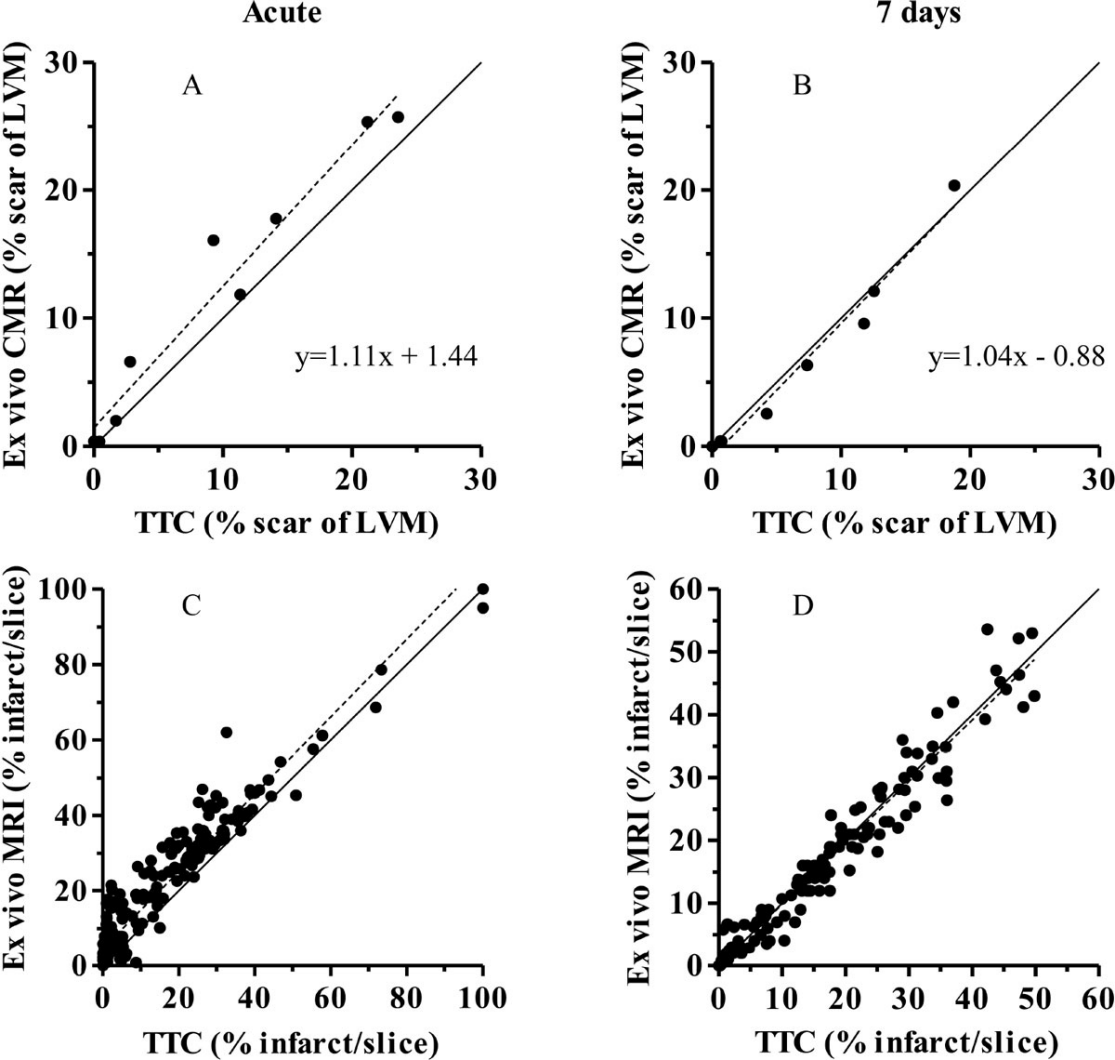
**Top panels show global infarct size by ex vivo CMR compared to TTC acute (A) and at 7 days (B)**. Bottom panels show a slice by slice comparison between ex vivo CMR and TTC acute (C) and at 7 days (D). Dashed line = linear regression and solid line = line of identity.

**Figure 2:**
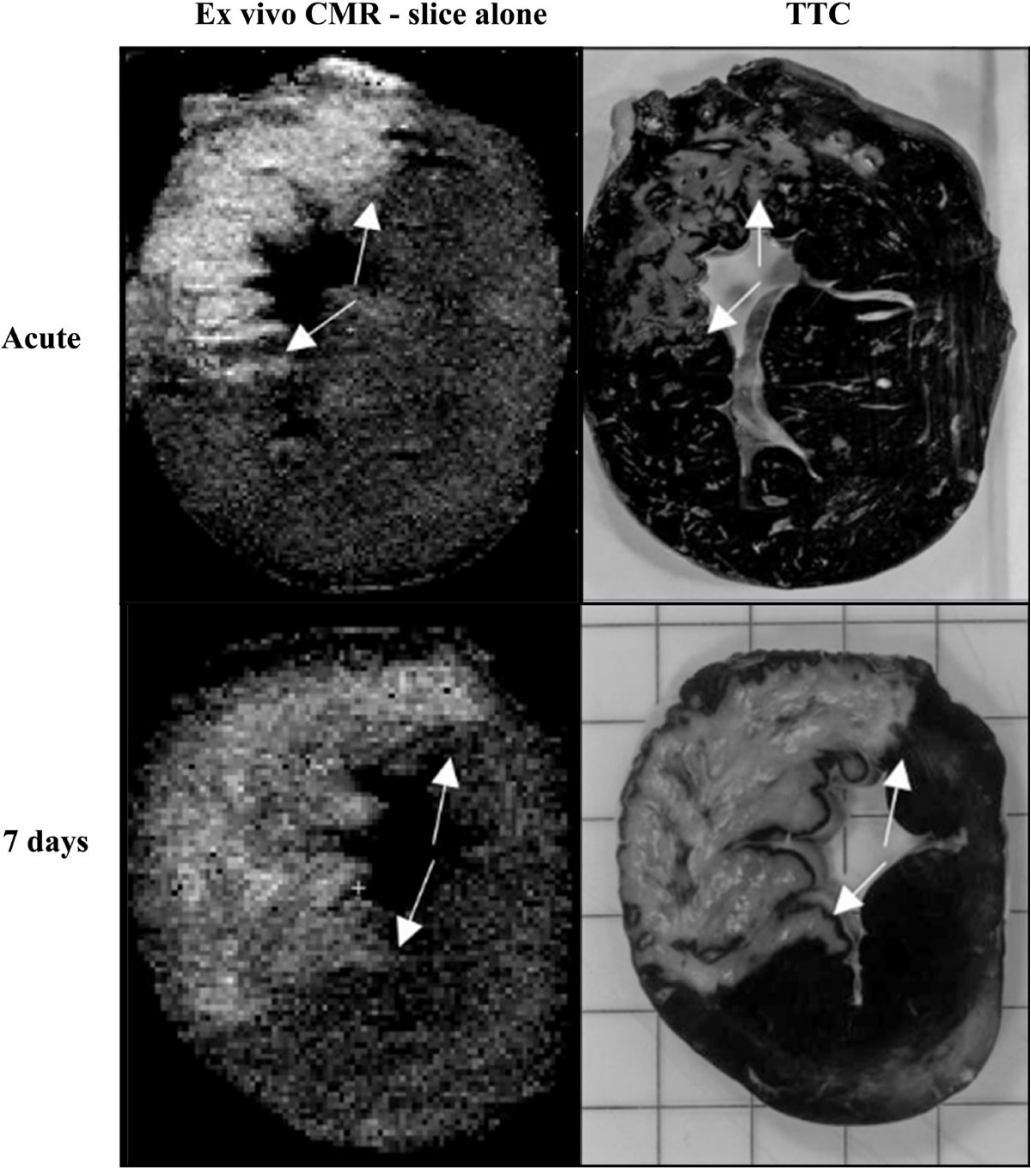
**CMR (left column) and TTC (right column) of ex vivo slices from pigs with acute infarction (top row) and from pigs 7 days after infarction (bottom row)**.

Infarct size (% of LVM) *in vivo *and *ex vivo *showed good agreement both acute and at 7 days (-0.012 ± 2.2%, p = 0.86 and 0.65 ± 1.2 vs, p = 0.63, respectively). The infarct size in the acute phase was overestimated by 11% with ex vivo CMR compared to TTC (11.8 ± 3.3% vs 9.4 ± 3.0%, p = 0.0078, Figure 1). However, no significant difference was seen at 7 days (6.1 ± 2.3% vs 7.0 ± 2.4%, p = 0.16). Slice by slice comparison between ex vivo MRI and TTC also showed a significant overestimation at the acute stage but no significant difference at 7 days (bias acute: 4.9 ± 5.6%, p < 0.0001; bias at 7 days: -0.22 ± 2.7%, p = 0.28, Figure 2). A significant decrease in relative fDV ratio (unitless) was seen in the PiZ (fDV_PiZ_/fDV_remote_) at 7 days compared to the acute phase (2.4 ± 0.21 vs 1.5 ± 0.15, p = 0.038).

## Conclusions

There is an overestimation of MI size by CMR compared to TTC in the acute phase but not after 7 days following acute MI. This is associated with a significantly higher fDV in the peri-infarction zone in the acute phase compared to 7 days later, indicating recovery of reversibly injured myocardium.

## Funding

Swedish Research Council, Swedish Heart Lung Foundation, Region of Scania, Medical Faculty at Lund University.

